# User characteristics and service satisfaction of car sharing systems: Evidence from Hangzhou, China

**DOI:** 10.1371/journal.pone.0263476

**Published:** 2022-02-02

**Authors:** Mengwei Chen, Yilin Sun, E. Owen D. Waygood, Jincheng Yu, Kai Zhu

**Affiliations:** 1 School of Design and Architecture, Zhejiang University of Technology, Hangzhou, Zhejiang, China; 2 Polytechnic Institute, Hangzhou, Zhejiang, China; 3 College of Civil Engineering and Architecture, Zhejiang University, Hangzhou, Zhejiang, China; 4 Department of Civil, Geotechnical, and Mining Engineering, Polytechnique Montréal, Montréal, Canada; Univerza v Mariboru, SLOVENIA

## Abstract

Car sharing has become a new mode of transport during the past two decades in the world. Its rapid growth in China has attracted a wide range of users and posed some problems. The main focus is on service efficiency and user satisfaction. To explore possible service enhancement and management intervention, this study aims at capturing the user characteristics according to different user types and scrutinizing their satisfaction with station-based one-way car sharing service. The study firstly illustrates descriptive statistics of user profile. This is followed by a study of user satisfaction influenced by user rates on staffs, the efficiency of rental process, vehicle situation, the use of credit card and their familiarity towards rental station. Furthermore, by clustering users according to the total travel time and distance during one rent, two different types of users are identified and defined as User Group A (UGA) and User Group B (UGB). To examine how fully do users utilize the shared cars, ANOVA was conducted implying family car ownership, total travel distance and main travel purpose have strong impact on total rental time for UGB, while for UGA, travel purpose and age have strong impact. Finally, ordinal logistic regression was introduced to find that for UGB, “shopping” is the main travel purpose with longer rental time, whereas for UGA, “out for business”, “shopping”, “visit friends” or “pick up others” are the main travel purposes with longer total travel time. Based on the findings, advices for operators on how to improve service quality and suggestions for government management strategy are discussed, respectively.

## Introduction

During the past two decades, car sharing has become a mainstream mode of transportation [1], especially in metropolitan areas where such services grow at a tremendous speed [2]. By October 2016, car sharing had its members of 15 million all over the world, 8.7 million of which were from Asia [3]. Stemmed from 1940s in Europe, the program has come a long way to be brought to Asia in the 1990s, ten years after it first gained popularity in Switzerland and Germany [4–6]. The adoption of smartphone based technology turns car sharing into one of new mobility-on-demand services [7]. The most common service type is station-based one-way car-sharing service which allows users to rent cars at stations and return them at any stations after short journeys [8]. The emergence of free-floating car sharing service attracts more users [9], due to the flexible rules that the vehicle can be returned to any location within its operational area [10]. For users, car sharing service reduces mobility costs [11], while for the society, it cuts down significant CO_2_ emission [12, 13], private car usage [10], energy consumption [11] and traffic congestion [14]. As a promising mode of travel, it is regarded as a supplement not only to public transportation, but to private mobility as well [5].

Car sharing is also booming and growing rapidly in China [15]. According to iMedia Research, car sharing has reached a market size of 430 million CNY till the end of 2016 [16]. At the policy level, the Chinese government is supportive of car sharing for new energy resources concerns [17]. Local government has implemented schemes involving technical research, parking facility support and pilot project start-ups. A report carried out by Roland Berger consultancy indicating that there is a huge gap between the number of driver’s license holders and the number of private car ownership, implying a potential market for car sharing in China in the coming years [18]. With the development of electric cars, most of the car sharing programs use electric cars to support the use of green energy.

Evidence show that car sharing is of great importance to reducing private car usage and energy saving [19]. Despite the promising future, there are still issues that are important to the change in citizens’ perceptions from a car property vision to a car-as-a-service one [20]. Few research focused on the efficiency of service usage from the perspective of user behavior. Judging from our preliminary interviews with users and operators, efficient service of car sharing was essential for a sustainable car sharing service. This can be achieved if users make full use of shared cars when rent it. Some users rented a car and kept it parked, while some users wanted to rent a car but no available ones. However, existing research pays less attention on it. Factors affecting the extent to which users use car sharing services lacks specific survey investigation and analysis. In addition, the impact of overall user satisfaction toward car sharing has not been examined from the perspective of each service aspect.

This study presents a modest effort to narrow these gaps. First, it aims to provide some empirical evidence to interpret the outcomes of users’ overall satisfaction in terms of user satisfaction with specific service aspects. Second, it attempts to reveal the major determinants of carsharing service usage based on a cross-sectional survey by distinguish different groups of users and scrutinizing their characteristics and travel behaviors.

The rest of the article is organized as follows. The second section presents a literature review. The third section describes the data and methods. The fourth section presents the empirical results and discussion. The fifth section addresses implications. Finally, the last section concludes.

## Literature review

### Service enhancement

While car sharing is rapidly developed, literature has seen quite a few studies focusing on service enhancement. By developing decision-supporting models, these research outcomes helped with optimal locations determination and vehicle relocation for the system. Kek et al. drew their attention to the reduction in staff cost, vehicle unavailable time and relocations and suggested a simulation model to enhance the operational efficiency for each unique station-based system with operating parameters [21]. Later on, by observing one-way car sharing, Correia and Antunes proposed an optimization approach to examine vehicle stock imbalance and found out that 22.7 vehicles per 100 trips is the minimum relocation operations [22]. Brandstätter et al. introduced a two-stage stochastic optimization problem to decide station distribution of electric car sharing systems, aiming at reducing traffic pollution, congestion and fossil fuel consumption [23]. With the recent technology development of Vehicle-to-Grid, Caggiani et al. focused on its application to electric carsharing system and proposed a model to relocate vehicles every morning according to the overnight vehicle distribution [24]. Research also covers free-floating systems where Weikl and Bogenberger presented a practice-ready relocation model of both conventional and electric vehicles to increase the profit for operators [25].

From the perspective of operators, Schmöller et al. used spatiotemporal analysis and implied there is an asymmetry between rental demand and supply on Monday that necessary actions from operators need to be taken for vehicle relocation [26]. Boyaci et al. then argued that for one-way non-floating systems, ensuring vehicle availability and reservation completion is prominent to meet the need of imbalanced demand [27]. As electric car sharing was introduced, Benarbia et al. developed an agent-based relocation model in a stochastic Petri Nets to help operators to reduce the relocation costs and attract more customers [28].

Few research payed attention to user satisfaction of car sharing service when discussing service enhancement, except that Silva and Schouery proposed mixed-integer programming models to maximum customers’ satisfaction by optimizing car assignment [8]. However, evidence show that latent attitudes of costumers on car sharing services were of great importance to affect car-sharing choice [29, 30]. Therefore, user satisfaction should receive more attention. By discovering the influencing factors and optimizing them, we can essentially improve the service quality.

### User characteristics

Another virtual focus in the research field is service utilization. Existing research shows a great interest in user characteristics. One of the major objectives is to explore the potential market. Kim et al. found that individual latent attitudes towards short-term day-to-day car sharing decisions were negatively affected by time constraints, lack of spontaneity and unpredictable travel times [31]. From the viewpoint of psychological effect on behavior, Curtale et al. found that social influence is the most important factor that effect car sharing acceptance [29].

For the existing market, several common characteristics were revealed. Results showed that users are tent to be young and highly educated who live or work in higher-density areas such as city centers [2, 7]. Kim et al. insisted the changes of car ownership be achieved not only by the improvement of vehicle itself, but also by customer education [32]. As for user income, Dias et al. also mentioned that higher-income is a feature of car sharing participants [7], while Efthymiou and Antoniou explained that users of low to medium income have a higher probability to join the program [33]. Tao et al. discovered that individual’s housing condition and income influence their choice of car sharing [19]. Apart from this, Prieto et al. found male users are more representative [2], which is constant with the results found by Ceccato and Diana [34].

Moreover, user travel behavior was also scrutinized by studies. In terms of mode of travel, Efthymiou et al. pointed out car sharing is more welcomed to people who used to commute by bus, trolleybus or tram [35]. With the aim of searching the difference and similarities between car sharing members and non-members in multimodal pattern, Kopp et al. discovered the former are more intermodal and multimodal in travel behavior; and shares of cycling are significantly higher whereas shares of private car trips are lower compared to the latter [36]. By examining the differences of user choices between carsharing services and traditional travel modes, Carrone et al. found that free-floating car sharing is a strong competitor of public transport in mode substitution [37].

Although there is few study focusing on service usage efficiency, service utilization has been noticed in present years. Wang and Liao proposed two disaggregate “first-come-first-serve” mechanisms to improve the utilization of shared cars [38]. Alencar et al. Found that compared with station-based one-way car sharing or free-floating car sharing service, station-based two-way service is of low utilization [39]. These findings provided new research angle, which gave clues to our study of discovering the factors that influence service efficiency from the view of user characteristics and travel behavior.

## Data and methods

### Survey design and data collection

Based on random sampling method, data were drawn from intercept survey in the rental stations to find out how the users felt about the service and to understand the characteristics about them. The study was conducted in Hangzhou, for it had the longest operating history whose experience could be referred to by other cities. As one of the earliest domestic cities introducing car sharing program, Hangzhou initiated electric station-based one-way car sharing in 2013 in the country and rapidly gained large market. By the end of August 2015, three months after the survey was conducted, 13,000 vehicles and nearly a hundred stations were in operation. To conduct intercept survey, eight stations were selected. They were located at areas of different land use including a residential area, a scenic spot, a business district, a bus terminal, a stadium, a university, a metro station (near shopping a center) and an industrial park.

The survey focused on a series of questions concerning three parts, including user personal attributes, their experiences with car sharing service and their satisfaction level towards the service. Questions related to user personal attributes and latest rental experience were developed to collect Person Trip (PT) data, while questions concerning user satisfactions were used to collected Revealed Preference (RP) data. Therefore, PT data was adopted to examine characteristics and behavior factors that influenced service usage, while RP survey provided data for user satisfaction analysis.

The survey was run from 13th to 20th in May, 2015, covering work days and weekends. Investigators conducted the survey by asking customers to answer all the questions in the questionnaire anonymously after they came returning the cars at each stations. If respondents provided information beyond what was included in the questionnaire, investigators would record it. Finally, a total of 129 respondents gave effective feedbacks, forming the research samples.

### Data analysis method

Data analysis is consist with three parts using mainly descriptive analysis, analysis of variance (ANOVA), clustering and ordinal logistic regression. The three parts of the analysis corresponded to three questions: a) who are using the car-sharing service? b) does the service of car sharing satisfied customers? and c) what are their characteristics that influence service usage?

#### Descriptive analysis

The first part of results presented a descriptive analysis to introduce the data profile. This section gave a rough description of samples.

For user attributes, sex, age, education background, career, family car ownership, monthly income were included. As for trip attributes, variables consisted of total travel time, total rental hours, main travel purpose and willingness to rent again. In context of satisfaction, overall satisfaction was asked, as well as user satisfactory level towards staff in the station, time spent during the rental procedure, car quality, the use of credit card, and their knowledge of station distribution.

#### User satisfaction analysis

To discover user satisfaction of the service, the second part conduct ANOVA to illustrate significant factors. In the study, Users’ overall evaluation of car sharing service is the dependent variable. Independent variables were user satisfactory level with car quality, staff in the station, time spent during the rent use of credit card and their familiarity of rental stations.

The aim of applying ANOVA was of two points. Most importantly, it was conducted to examine significant factors influencing user satisfaction of car sharing. The independent variables selected were specific service aspects, whose significance was a direct indication of its importance in the overall service. Moreover, the values of estimates for significant variables gave proves to show the order of their importance.

#### Analysis of factors influencing service usage

In the third part, factors influencing service usage was examined. There were three steps of the analysis. First, users were classified into different groups. Hypothetically, in order to make full use of the service, it is preferable for car sharing service to maintain a high turnover rate. This means users spent the majority time driving the car after renting. However, different users might have different behavior. Therefore, this study adopted clustering to divide users into groups according to different level of service usage. Total travel distance and rental time were selected as indicators. The clustering adopts log-likelihood as the distance measure. To prevent from over fitting, Bayesian Information Criterion (BIC) was adopted to help decide the best number of clusters. The equation is as follow:

BIC(J)=−2ln(L)+kln(n) (1)

dBIC(J+1)=BIC(J+1)−BIC(J). (2)

where, *J* is number of clusters, *L* is the likelihood function and n is the sample size. Here, the number of clusters with the largest change from the previous one is chosen as the most proper number of clusters. Note that *d* in the equation denotes derivative. Finally, users were divided into two groups.

After classifying user groups, the second step applied ANOVA to scrutinize user characteristics among age, family car ownership, total travel distance and main travel purpose of each group. Total rental time was chosen as dependent variable to reflect car usage. According to the results, travel purpose was significant in both analysis, it was selected to be further studied in the next step.

Finally, in the third step, the study applied ordinal logistic regression to take a look at specific travel purpose of different user groups that mattered to service usage and made a comparison. The dependent variable was total rental time and travel purpose was the independent variable. Ordinal logistic regression analysis model is explained as follow:

$$Y=\ln\frac{\pi_{ij}(Y\leq j)}{1-\pi_{ij}(Y\leq j)}=\ln\frac{\pi_{i1}+\cdots +\pi_{ij}}{\pi_{i(j+1)}+\cdots +\pi_{iJ}}=\alpha_{j}-(\beta_{1}X_{i1}+\cdots +\beta_{n}X_{in})$$ (3)

where *i* represents the level of each independent variable; *j* represents the level of dependent variable; *α* is the constant; and *β* is the estimate parameter. The logarithms of the estimated parameter are used to show the levels precede the reference level. Note that ln in the equation denotes logarithm.

## Results and discussion

### Who are using the car-sharing service?

Descriptive analysis gives an overview of the participants and their journeys accomplished by shared cars. Thus, the results have answered the question of “Who are using the car-sharing service?”. In detail, gender, age, family car ownership, monthly income, user occupation and education background are investigated as user attributes, while total rental hours, total travel distance during one rent, main travel purpose and willingness to rent again were examined as trip related variables.

Results listed in **Table 1** illustrate that males are the main users, occupies 84% of the total car sharing population. This corresponds to the finding proposed by Dias et al. [7]. It may be due to the fact that male drivers’ license holder exceeds female in most of the Chinese cities. Most of the users are at the age between 25 and 34. Among them, 25 to 29 has the largest shares of 49% while users above 40 years old form less than 5%. The numbers show the probability that the younger generation has different attitude to car use compared to the older generation [2]. Moreover, this group of people just begins working who cannot afford to purchase a car, yet they still have the needs of using it. This may be proved by the statistic of family car ownership to a certain extent, that is, the largest group of users (76%) does not have a car. It is interesting to notice that 20% of the users are one-car owners who also participate in car sharing program, partly because of the policy intervention that every gasoline car need to be off the road during the peak hours for one work day but electric cars do not. However, some of these users really need to drive on that day. Besides, although non-car owners take more than three quarters, users with one car or more still come renting. Many of them admit they are coming to the city for business reasons and car sharing is their best choice. From the results of income distribution, most of the respondents earn more than 5,000 CNY a month, suggesting an upper middle level of salary. As for job information, users working for private enterprises takes more than half of the group, followed by workers from foreign-funded enterprises (12%) and self-employed individuals (12%). Flexible working hours, higher wages and needs of efficient travel may explain the reasons why they are the biggest part of car sharing members. Some of them are neither locals nor decide to settle down in Hangzhou. These findings match mostly with the previous work provided by Wang et al. to study the potential users in a Chinese city [40]. As for education background, 95% of users have bachelor degree, which is consistent with findings of Prieto et al. [2].

**Table 1:** Descriptive statistics of users.

Variable	Category	Percent (%) or Mean (SD)
**Socio-demographics**		
Gender	Male	83.8
	Female	16.2
Age	18–24	10.9
	25–29	49.2
	30–34	28.1
	35–39	7
	40–44	3.1
Family car ownership	None	76.2
	1 car	20
	2 cars or more	3.8
Monthly income (CNY)	0–1999	5.4
	2000–4999	30.2
	5000 or more	64.3
Occupation	Government sector	4.6
	State enterprise	7.7
	Private enterprise	52.3
	Foreign-funded enterprise	11.5
	Self-employed	11.5
	Retired	0
	Student	5.4
	Unemployed	1.5
	others	5.4
Education background	Junior High School Degree or lower	0.8
	High school/ junior college degree	3.8
	Bachelor degree	83.1
	Master/PhD degree	12.3
**Trip characteristics**		
Total travel distance	Less than 10km	6.2
	10km-20km	22.5
	20km-30km	21.7
	30km-40km	27.9
	40km-50km	9.3
	50km-60km	7
	60km or longer	5.4
Total rental hours	0-59m	9.3
	1h-1h59m	23.3
	2h-2h59m	27.1
	3h-3h59m	20.9
	4h-4h59m	9.3
	5h-5h59m	3.9
	6h-6h59m	1.6
Main travel purpose	Commute	0.8
	Go home	2.3
	Out for business	18.6
	Shopping	7.8
	Leisure	41.1
	Visit friends	3.1
	Pick up people	10.1
	others	16.3
willingness to rent again	Yes	97.7
	No	2.3
**User satisfaction**		
Overall satisfaction (1–5; 1 = very satisfied, 5 = very unsatisfied)		1.70 (±0.76)
Satisfaction for staffs (1–5; 1 = very satisfied, 5 = very unsatisfied)		1.53 (±0.66)
Familarity for rental stations (1–5; 1 = very familiar, 5 = don’t know at all)		2.70 (±1.02)
Satisfaction for efficiency of rental procedures (1–5; 1 = very satisfied, 5 = very unsatisfied)		2.00 (±0.81)
Satisfaction for vehicle situation (1–5; 1 = very satisfied, 5 = very unsatisfied)		2.38 (±0.81)
Satisfaction for credit card use (1–5; 1 = very satisfied, 5 = very unsatisfied)		2.74 (±1.02)

sample size: 129

PT data also shows some findings of trip related outcomes. Of all the short-term rents, the largest group is the ones whose total rental hours are below five hours, covering over 90% of time duration during the lease. Two to three hours are the highest frequency of vehicle rental time with shares of 27%. One to two hours ranks the second and three to four hours is the third with a percentage of 23% and 21%, respectively. Accordingly, the most frequent total travel distance is between 10km and 40km, showing a tendency of relationship between rental time and travel distance. Among eight main travel purposes, leisure is the first common one, for the city has a scenic region of 49 km^2^ that active travel can hardly finish every spot. Besides, out for business is the second highest frequency of purpose, possibly because the low efficiency of public transport service and unstable accessibility to taxis or ride hailing service that failed to meet these people’s needs, especially in peak hours or taxi handover times. In all, 98% of the users address the willingness to use car sharing service again.

### Does the service of car sharing satisfied customers?

Now that members of car sharing have a high willingness to use the service again from the previous analysis, does that mean they are satisfied with it? By calculating the descriptive statistic of RP data presented in **Table 1**, a high level of positive feedback on the service was reported. In fact, user satisfaction strongly influences their car-sharing decision [41]. From this point of view, ANOVA is conducted to scrutinize the impact made by five factors on the users’ overall satisfaction of the service.

As illustrated in **Table 2**, car-sharing members’ rates on staff, rental station, the efficiency of rental procedures, vehicle situation and the use of credit card to rent and pay are all significant to the level of satisfaction with the whole service. All the independent variables are significant and the value of Sum of Squares indicates the effect of independent variable on the dependent variable. Of all the five factors, satisfaction for staffs has the largest sum of squares, indicating how the staff perform will severely impact the users’ feelings. This corresponds to the findings by Mavlutova et al. that responsive service enhance the service quality that attracts more users [42]. Moreover, vehicle situation is also of great importance whose sum of squares rank the second, for it directly linked with driving safety and comfort. In addition, the time taken during the rental procedures also has impact on user satisfaction, but not as strong as the previous two variables. Combined with survey results that the average time spent is less than 5 minutes, users find it acceptable as they expect. As for the familiarity of rental station distribution, respondents expressed that they had strong need for location indications so that they could better plan their trip using the service. Finally, for the use of credit card, they found it convenient and reasonable but it took a bit long to refund the deposit.

**Table 2:** Results of ANOVA on user satisfaction.

Variable	Sum of Squares
Corrected model	50.940^a^***
Intercept	54.439***
Satisfaction for staffs (1–5; 1 = very satisfied, 5 = very unsatisfied)	16.398***
Familiarity for rental stations (1–5; 1 = very familiar, 5 = don’t know at all)	2.451**
Satisfaction for efficiency of rental procedures (1–5; 1 = very satisfied, 5 = very unsatisfied)	2.938***
Satisfaction for vehicle situation (1–5; 1 = very satisfied, 5 = very unsatisfied)	4.942***
Satisfaction for credit card use (1–5; 1 = very satisfied, 5 = very unsatisfied)	2.404**
Error	22.177
Total	441
Corrected total	73.117

note: Na = not applicable.

*** Significant at α = 0.01

** Significant at α = 0.05.

^a^R^2^ = 0.697.

### What are the user characteristics influencing service usage?

After exploring the satisfaction of the customers, research went further to study service usage by observing characteristics of these customers including individual attributes and their travel behavior. During the survey, one thing aroused users’ attention was the adequacy of shared cars. Since the service is basically charged by time within a certain distance set by operators, theoretically, users should use it as efficient as they can. Based on this assumption, clustering is conducted. Two variables, total travel distance and total rental hours, were selected and clustered.

**Fig 1:**
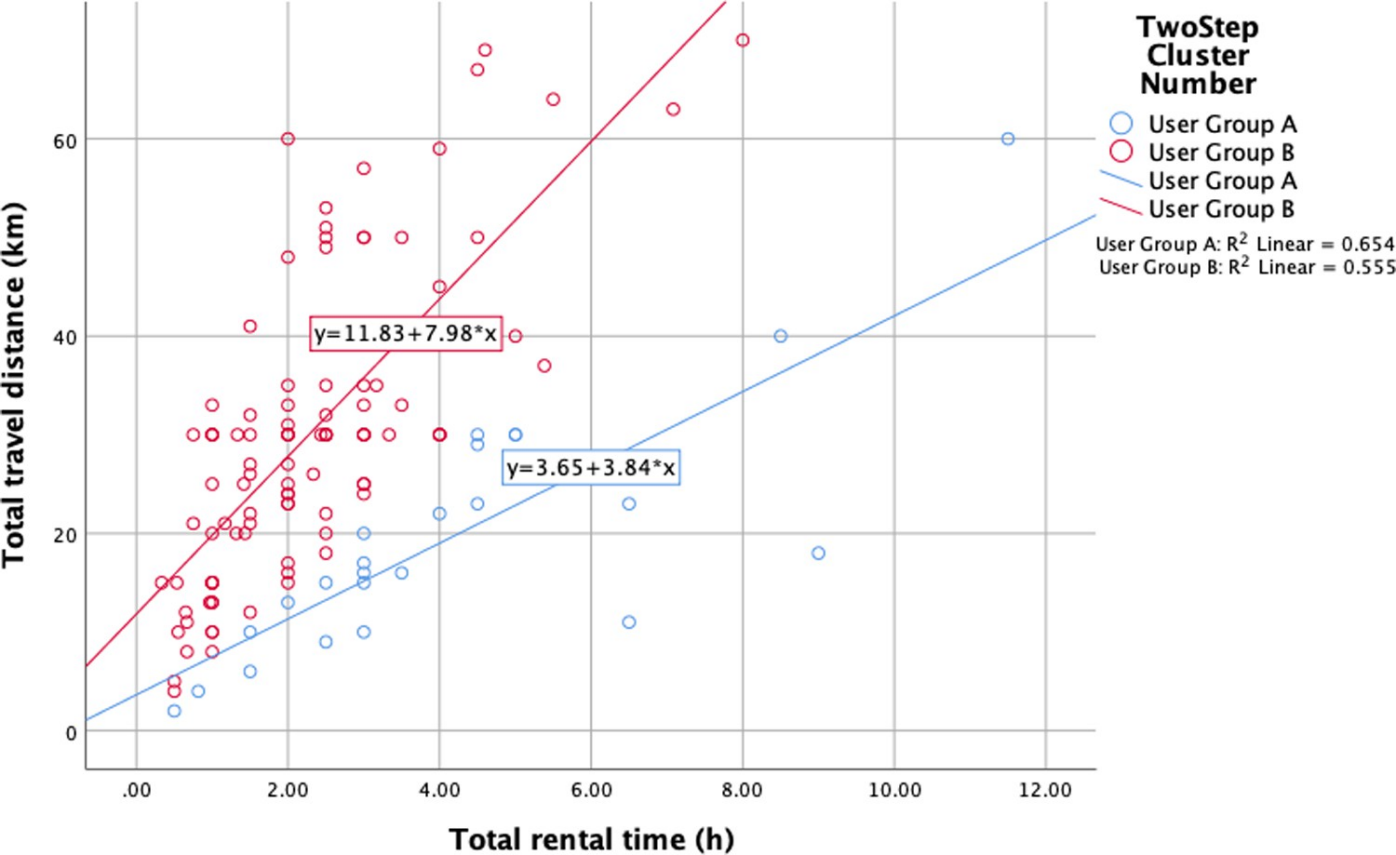
Scatter diagram of user clusters.

According to the rule of BIC, the data was classified into two clusters (**Table 3**) because the biggest change in BIC is in the row of 2 clusters. As described in **Table 4**, cluster distribution of the two have the shares of 19% and 81%, respectively. More visualized scatter diagram is displayed in **Fig 1** which reveals user features. From the linear fitting in **Fig 1**, cluster with 24 samples are defined as User Group A (UGA) who have smaller ratio of travel distance and rental time, indicating less use intensity. In simple terms, these users may spend less time driving and more time keeping the cars parked, or have rather lower driving speed. Relatively, cluster with 105 samples are defined as User Group B (UGB) who have rather the opposite features.

**Table 3:** Auto clustering information of rented car usage.

Number of Clusters	Bayesian Information Criterion (BIC)	BIC Change^a^
1	98.635	
2	57.785	**-40.850** ^**b**^
3	49.200	-8.585
4	50.475	1.275
5	55.382	4.907

note

^a^ The changes are from the previous number of clusters in the table.

^b^ The biggest change in BIC.

**Table 4:** Cluster distribution of rented car usage.

Cluster	Number of Sample	Percentage (%)
1	24	18.6%
2	105	81.4%
Total	129	100.0%

From the data, two different group of users showed different tendency of how to use the vehicles. In order to know the cause behind the phenomenon, ANOVA is used to explore the characteristics of each type of users. Total rental time was used as the dependent variable not only because it directly reflected the time spent during the lease, but also was the main reference for rental charges.

From the results of ANOVA for UGB shown in **Table 5**, family car ownership, total travel distance and main travel purpose are the three key variables significantly influence the rental time. Among them, total travel distance has the largest impact, implying a close positive relationship between time and distance. It is reasonable probably because for UGB, they drove most of the time as they used the vehicle efficiently. Main travel purpose has the second strong influence on the dependent variable, giving the possibility that UGB might have rather specific aims for the trip which last not for so long. There are few clues can explain why family car ownership is significant, so the analysis in the following part will continue to dig up. Age is not significant, so it was not included in the model.

**Table 5:** ANOVA of UGB.

Variable	Sum of Squares
Corrected model	169.136^a^***
Intercept	26.507***
Family car ownership	4.535**
Total travel distance	140.534***
Main travel purpose	8.351**
Error	28.264
Total	758.924
Corrected total	197.400

note: Na = not applicable.

*** Significant at α = 0.01

** Significant at α = 0.05.

^a^R^2^ = .857.

Compared to UGB, ANOVA for UGA has different outcomes (**Table 6**). Having the same dependent variables, main travel purpose and age are the two significant explanatory variables. From the value of Sum of Squares, main travel purpose has the strongest impact on rental hours. It is interesting to notice that main travel purpose is also a significant factor for UGB. Thus, assumption can be drawn that different main travel purpose can be one of the reasons that lead to different rental behavior. Finally, to UGA, age is also a significant factor, which is a unique factor compared to UGB.

**Table 6:** ANOVA of UGA.

Variable	Sum of Squares
Corrected model	362115.39^a^**
Intercept	975566.187***
Main travel purpose	239833.166**
Age	125179.696*
Error	210661.221
Total	1921201.000
Corrected total	572776.609

note: Na = not applicable.

*** Significant at α = 0.01

** Significant at α = 0.05

* Significant at α = 0.1.

^a^R^2^ = .632.

As the main travel purpose is important variable for both UGB and UGA on the basis of the two groups of ANOVA results, further study focusing on more detailed features is required. To achieve this, ordinal logistic regression was adopted to analyze every item for “main travel purpose” for UGA and UGB, respectively.

Results explained in **Table 7** help selecting the detailed significant main travel purpose, pointing out that for UGB, shopping is the one that influence the time length for shared car use compared with the category of “others”. This is reasonable that compared to other purposes, shopping takes longer time so that the total rental time tend to be longer. Other purposes do not have significant impact on rental time. Unlike UGB, UGA have a variety of main travel purposes influencing rental time duration. Compared to the purpose “others”, four items are significant. “Pick up someone” has the highest value of estimate, showing a strong tendency of longer rental time. Perhaps the waiting time for the picked up guests can be unpredictable, especially when the delay occurs, the time will be wasted. Shopping and visiting friends also have a strong effect, as both activities usually require time spent making decisions and communicating with people. Finally, “out for business” is also a strong explanation for longer rental time, because this kind of activity requires efficiency on the road but takes considerable time when handling the business [43].

**Table 7:** Results of ordinal logistic regression on the main travel purpose influence the rental time of UGB and UGA.

Variable		Estimates	
		UGB	UGA
Main travel purpose	commute	0.204	Na
	Go home	-1.492	Na
	Out for business	-0.482	1.915**
	Shopping	1.568**	3.467**
	Leisure	0.549	0.586
	Visit friends	-0.287	3.462**
	Pick up someone	-0.643	3.467**
	others	0^a^	0^a^

note

^a^ This parameter is set to zero because it is redundant.

** Significant at α = 0.05

Na = not applicable.

In summary, our results suggest improvements to specific aspects of the rental service can enhance overall user satisfaction, especially the socialization of people in the rental process, this corresponds to findings of Curtale et al. [29]. Findings also suggest users with different travel purposes use shared cars with different intensities. The vehicle usage length tends to be closely linked to the activities related to the purpose of the trip. This can be the evidence for carsharing market segmentation, with different efficiency of service use according to users.

The research still has room for improvement. The survey was conducted two years after the electric car sharing service was born, with limited market penetration and data sample size. Cross-sectional data does not capture the change of user behavior and satisfaction. However, along with the emergence of self-service renting, this change may exist, so further research may be needed.

## Implications

This study provides some policy implications. First, our findings offer evidence to support the current efforts made by system operators to enlarge the market of electric car sharing service. According to satisfaction study, though with the help of smartphone apps, renting procedures are mostly done online, staffs’ performance still strongly affects the service quality. To improve this, instant access and open-ended booking service need to be guaranteed to save the manual works and make the service more flexible [44]. Additionally, car quality also strongly influence user satisfaction. In fact, a number of recent failures in car sharing operation have been due to poor car quality. It is important for the operators to weigh the cost of car acquisition against service charges and include user credit in the fee or deposit management. Moreover, since the location distribution of stations is the key issue that users cared about, and “shopping”, “out for business” are the travel purposes that needs longer time, the accessibility of stations near work place and residential areas can be increased by putting some self-service rental and parking stations. To achieve this, the support from government is crucial because it needs the room for parking with the facility for charging [45, 46]. From the statistical analysis, travel purposes are the most important factor influencing the rental time. Results showed that for both types of users, shopping is the main travel purposes that lengthen the rental time, so operators can open the advertisement business by setting a place for shopping guidance or coupons in the car for stores or brands to earn some profits. Also, longer journey time often accompanied with travel purposes linking to public places. Thus, setting big scale stations near residential places and dispersive and small-scale stations (or even self-service parking lots) in commercial districts can be a feasible solution. This may be an idea to attract both types of the users, as well as to mitigate the uneven distribution problem for one-way car sharing system.

Second, the findings provide some clues to help the government to optimize car sharing management strategy. At some degrees, decision makers may control or regulate car sharing development considering the joint adoption with other policy intervention. To improve the use efficiency of the services, UGB are the main targeted users and UGA are encouraged to use alternative traffic mode, especially the public transport, according to different main travel purposes. Short-distance journeys with the travel purpose of “out for business” and “visit friends” can be replaced by service like bikesharing [47].

Third, this study implies that more emphasis should be placed on the mechanism behind the development of car sharing service and its user needs. As electric car sharing has just started, its safety and battery’s state of health need to be ensured [48]. A good maintenance for the vehicles and sustainable battery disposal are necessary. With the help of smartphone, manual work can transfer from reception to background operation and system maintenance. From a broader perspective, with the development of autonomous driving, car sharing will have a better prospect and larger user market. Unlike driving behavior that will be replaced by technology, there is no substitute for user travel behavior. Therefore, this research is not only relevant to the operation and management of car sharing at this stage, but also provides a basis for studying the evolution of rental and travel behavior of car sharing.

## Conclusion

In conclusion, this paper has examined factors influencing user satisfaction service usage. Descriptive statistics depicted a clear picture that most of the users are male, at the age between 25 and 34, non-car owners and workers in private enterprises or foreign-funded enterprises or self-employed. Their trips by shared cars are mostly ranging from one to four hours and 10km to 40km. The most frequent main travel purpose is leisure. Of all the users, 98% of them confirm they are willing to use car sharing service again. The above information put together can answer the question of “who are using the car sharing service?”. Study of user satisfaction is carried out indicating how users rate the rental station, the staffs, the efficiency of rental process, vehicle situation and the use of credit card will significantly affect their satisfaction for the service. This explains “does the service of car sharing satisfied customers?”. By clustering users according to the time spent and distances travelled together during one rent, two different type of users defined as UGA and UGB were identified. To examine how fully do users take the use of the shared cars, ANOVA is conducted. Results implied that family car ownership, total travel distance and main travel purpose have strong impact on total rental time for UGB while for UGA, main travel purpose and age are significant variables. Ordinal logistic regression was introduced to further analyze the details of the main travel purpose influencing the rental time. The results showed that for UGB, users taking “shopping” as the main travel purpose tend to rent for longer time whereas for UGA, “out for business”, “shopping”, “visit friends” or “pick up someone” is the main travel purpose that needs longer billing hours. These analyses replied to the question of “what are the characteristics influencing service usage?”.

This study contribute to the literature in two aspects. First, it provides empirical evidence to examine the impact of users’ overall satisfaction in terms of user satisfaction with specific service aspects and user knowledge about the service facility. Second, it tries to reveal the major determinants of carsharing service usage based on survey data by identifying different groups of users and scrutinizing their characteristics and travel behaviors.

## Supporting information

S1 File Questionnaires. (ZIP)

S2 File Research data. (ZIP)
