# Supplementary material for: User characteristics and service satisfaction of car sharing systems: Evidence from Hangzhou, China
Source: PLoS One. 2022 Feb 2;17(2):e0263476. doi: 10.1371/journal.pone.0263476 (PMC8809597; doi:10.1371/journal.pone.0263476)
Supplement: S1 File — (ZIP) [file pone.0263476.s001.zip › S1 Questionnaires/PT survey-Chinese version.pdf]

微公交出行调查问卷

亲爱的用户，您好！为了了解杭州市微公交的出行情况，我们需要对微公交的使用人群进行问卷调查。然后根据调查结果通过统计分析等方法，找到目前微公交出行存在的问题，分析原因以及提出解决对策，为杭州市微公交的发展献计献策，方便市民的出行。请您尽可能写得详细点。问卷采用匿名方式作答，您无需填写姓名，所得数据仅供研究之用，敬请放心。占用了您的宝贵时间，向您致以深切的歉意和谢意！您的配合和支持是我们调查成功的关键，祝您身体健康、工作顺利！

调查日： 2015年 ＿＿＿月 ＿＿＿日 调查员编号：

调查站点：		被调查人编号：	个 人 特 征 表								
**1.性 别**	**2.年龄（岁）**	**3.受教育程度**	**4.职 业**	**5.杭州市可用家庭小汽车保有量**	**6.月经济收入(元)**	**7.驾龄**	**8.外出经常采用的交通方式（可多选）**	**9.家至最近微公交站点步行时间**	**10.工作地至最近微公交站点步行时间**	**11对微公交的了解程度**	**12是否会再次租赁微公交**
① 男<br>② 女	＿＿ 岁	① 小学或以下<br>② 初中<br>③ 高中、中专<br>④ 大学<br>⑤ 研究生	① 国家行政机关、事业单位<br>② 国营企业 ③ 民营企业 ④ 外资、合资企业<br>⑤ 个体经营户<br>⑥ 退休 ⑦ 学生<br>⑧ 无业 ⑨ 其他	①0辆<br>②1辆<br>③2辆<br>④3辆及以上	① 0～1999<br>② 2000～2999<br>③ 3000～3499<br>④ 3500～3999<br>⑤ 4000～4999<br>⑥ 5000及以上	① 1年以下<br>② 2～5年<br>③ 5～10年<br>④10年以上	① 步行 ② 自行车<br>③ 电瓶车 ④ 公交车<br>⑤ 地铁 ⑥ 出租车<br>⑦ 小汽车 ⑧ 班车<br>⑨ 公车 ⑩ 其他	① <5分<br>② 5～9分<br>③ 10～19分<br>④ 20～29分<br>⑤ >30分	① <5分<br>② 5～9分<br>③ 10～19分<br>④ 20～29分<br>⑤ >30分	① 非常熟悉<br>② 一般<br>③ 不熟悉	① 会<br>② 不会

个 人 出 行 情 况 调 查 表															
次 数		**出 发**					**到 达**			**一次出行过程**					
		租车时间		出发站点	是否有公共交通衔接	出行目的（请填写代号）	还车时间		到达站点	耗 时(分)				出行距离（km）	租车费用（元）
		时	分	＿＿＿＿＿＿＿＿＿＿			时	分	＿＿＿＿＿＿＿＿＿＿	到微公交站点时间	租车耗时	车上时间	还车耗时		
请按次序填写被调查日调查时刻前24小时所有微公交出行	1														
	2														
	3														
	4														
	5														
	6														
	7														

注：出行目的代码：1.上班 2.上学 3.回家 4.业务 5.回单位（学校） 6.购物 7.休闲娱乐 8.文艺体育活动 9.探亲访友 10.接送他人 11.其他；

是否有公共交通衔接：1.是 2.不是
