# Supplementary material for: User characteristics and service satisfaction of car sharing systems: Evidence from Hangzhou, China
Source: PLoS One. 2022 Feb 2;17(2):e0263476. doi: 10.1371/journal.pone.0263476 (PMC8809597; doi:10.1371/journal.pone.0263476)
Supplement: S1 File — (ZIP) [file pone.0263476.s001.zip › S1 Questionnaires/Satisfaction survey-Chinese version.pdf]

微公交的使用情况满意度调查问卷

1 您对本次微公交出行的满意度

①非常满意

②满意

③一般

④不满意

⑤非常不满意

2 您对工作人员的满意度

①非常满意

②满意

③一般

④不满意

⑤非常不满意

3 您对微公交站点分布的了解程度

①非常了解

②了解

③一般

④不了解

⑤非常不了解

4 您对租还车耗时程度的满意度

①非常满意

②满意

③一般

④不满意

⑤非常不满意

5 您对车辆情况的满意度（包括动力、续航、刹车系统及损毁情况等）

①非常满意

②满意

③一般

④不满意

⑤非常不满意

6 您对租车使用信用卡扣除 1000 元订金后十五个工作日返还收费方式的满意度

①非常满意

②满意

③一般

④不满意

⑤非常不满意

7 您认为本次租车还存在的其他问题及提出的建议
