# Supplementary material for: User characteristics and service satisfaction of car sharing systems: Evidence from Hangzhou, China
Source: PLoS One. 2022 Feb 2;17(2):e0263476. doi: 10.1371/journal.pone.0263476 (PMC8809597; doi:10.1371/journal.pone.0263476)
Supplement: S1 File — (ZIP) [file pone.0263476.s001.zip › S1 Questionnaires/Satisfaction survey-Englishi version.pdf]

User satisfaction survey on car sharing serivce

1 How are you satisfied with car sharing service on the whole?

① Very satisfied
② Satisfied
③ Neutral
④ Not satisfied
⑤ Very unsatisfied

2 How are you satisfied with staff in the station?

① Very satisfied
② Satisfied
③ Neutral
④ Not satisfied
⑤ Very unsatisfied

3 On what level do you know about car sharing service?

① Very familiar
② familiar
③ Neutral
④ Don’t know much
⑤ Don’t know at all

4 How are you satisfied with the time spent of the rental process?

① Very satisfied
② Satisfied
③ Neutral
④ Not satisfied
⑤ Very unsatisfied

5 How are you satisfied with car quality (including power, endurance, braking system, damage related situation etc.)?

① Very satisfied
② Satisfied
③ Neutral
④ Not satisfied
⑤ Very unsatisfied

6 How are you satisfied with the way the car rental charges are refunded in fifteen days after the credit card is used to deduct the deposit of 1,000 RMB?

① Very satisfied
② Satisfied
③ Neutral
④ Not satisfied
⑤ Very unsatisfied

7 Is there any other problems that you think is critical or do you have any suggestions for the service?
