# Supplementary material for: User characteristics and service satisfaction of car sharing systems: Evidence from Hangzhou, China
Source: PLoS One. 2022 Feb 2;17(2):e0263476. doi: 10.1371/journal.pone.0263476 (PMC8809597; doi:10.1371/journal.pone.0263476)
Supplement: S1 File — (ZIP) [file pone.0263476.s001.zip › S1 Questionnaires/PT survey-English version.pdf]

Travel survey on car sharing

Dear users:
In order to understand the situation of car sharing in Hangzhou, we are now conducting a questionnaire survey of users. Based on the results of the survey, we are to find the problems of car sharing service and the causes behind it using statistical models. In addition, we are going to propose advices for the development of car sharing in Hangzhou. Please provide as much details as possible. The questionnaire is anonymous, so you do not need to fill in your name. The data obtained is for research purposes only. Thank you for your valuable time and participation! Your cooperation and support is the key to the success of our survey. Wish you all the best!

Date: **2015/** (month)/ (day) **Investigator No:**

**Station:**		**Respondent No:**	**Personal Attributes**								
**1.Gender**	**2.Age**	**3.Education bachground**	**4.Career**	**5.Family car ownership**	**6.Monthly income(Yuan)**	**7.Length of driving experience**	**8.Major daily travel mode (multiple)**	**9.Walking time from home to the nearest station**	**10.Walking time from workplace to the nearest station**	**11.knowledge of car sharing**	**12.willingness to rent again**
① male<br>② female	___	① Primary Education<br>② Junior High School Degree<br>③ High school/ junior college degree<br>④ Bachelor degree<br>⑤ Master/PhD degree	① State administrative organs/institutions<br>② State-Owned Enterprises<br>③ Private enterprises<br>④ Foreign-invested/joint ventures ⑤ Self-employed<br>⑥ Retired ⑦ Student<br>⑧ Unemployed ⑨ Others	① None<br>② 1car<br>③ 2 cars<br>④ 3 cars or more	① 0～1999<br>② 2000～2999<br>③ 3000～3499<br>④ 3500～3999<br>⑤ 4000～4999<br>⑥ 5000 or more	① 1 year or less<br>② 2～5 years<br>③ 5～10 years<br>④ 10 years or more	① Walk ② Bicycle<br>③ E-bike ④ Bus<br>⑤ Metro ⑥ Taxi<br>⑦ Car ⑧ Unit shuttle<br>⑨ Unit assigned car<br>⑩ Others	① <5 min<br>② 5～9 min<br>③ 10～19 min<br>④ 20～29 min<br>⑤ >30 min	① <5 min<br>② 5～9 min<br>③ 10～19 min<br>④ 20～29 min<br>⑤ >30 min	① Very familiar<br>② Muetrual<br>③ Not farmiliar	① will rent again<br>② will not rent again

**Person-Trip**															
Trip number		**Departure**				Travel purpose (please fill in the code)	**Arrival**			**Information about one trip**					
		Rent time		Departure station ______	Whether shift to or from public transport		Return time		Arrival station ______	Total time (min)				Total travel distance (km)	Cost (Yuan)
		hour	min				hour	min		Time arrive at station	Time spent on rental	Time spent on the car	Time spent on return		
Please fill in the form about each trip using car sharing in order. Calculated within 24 hours before the moment of the survey.	1														
	2														
	3														
	4														
	5														
	6														
	7														

Note: Travel purpose code: 1.commute 2.go to schoole 3.go home 4.out for business 5.back to school/workplace 6.shopping 7.leisure 8.Cultural and Sports Activities 9.vist friends 10.Pick up and drop off others 11.others;

Whether shift to or from public transport: 1.Yes 2.No
