# Supplementary figures and images for: User characteristics and service satisfaction of car sharing systems: Evidence from Hangzhou, China
Source: PLoS One. 2022 Feb 2;17(2):e0263476. doi: 10.1371/journal.pone.0263476 (PMC8809597; doi:10.1371/journal.pone.0263476)

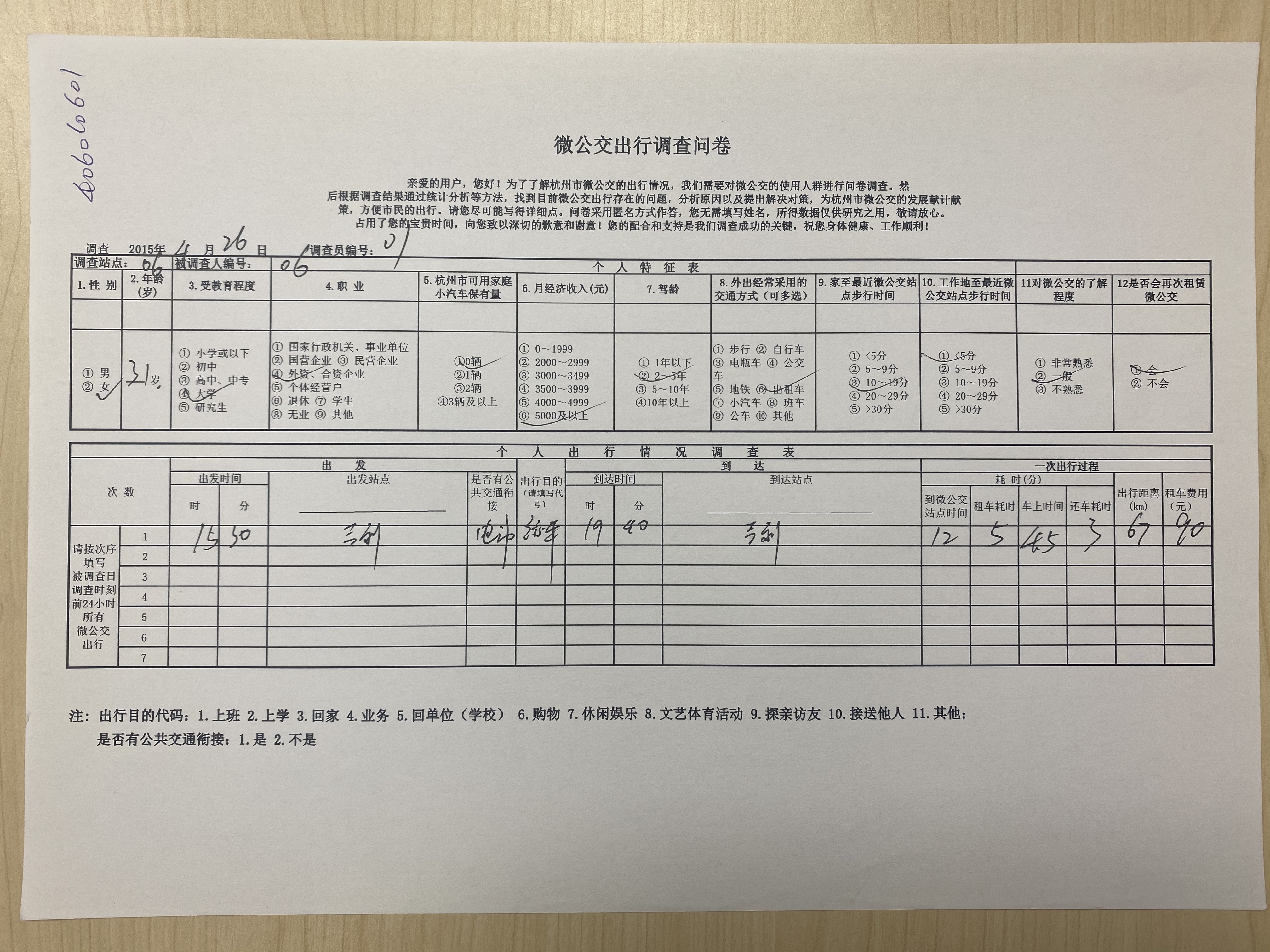

Supplement: S1 File — (ZIP) [file pone.0263476.s001.zip › S1 Questionnaires/PT survey-Sample 1.jpeg]

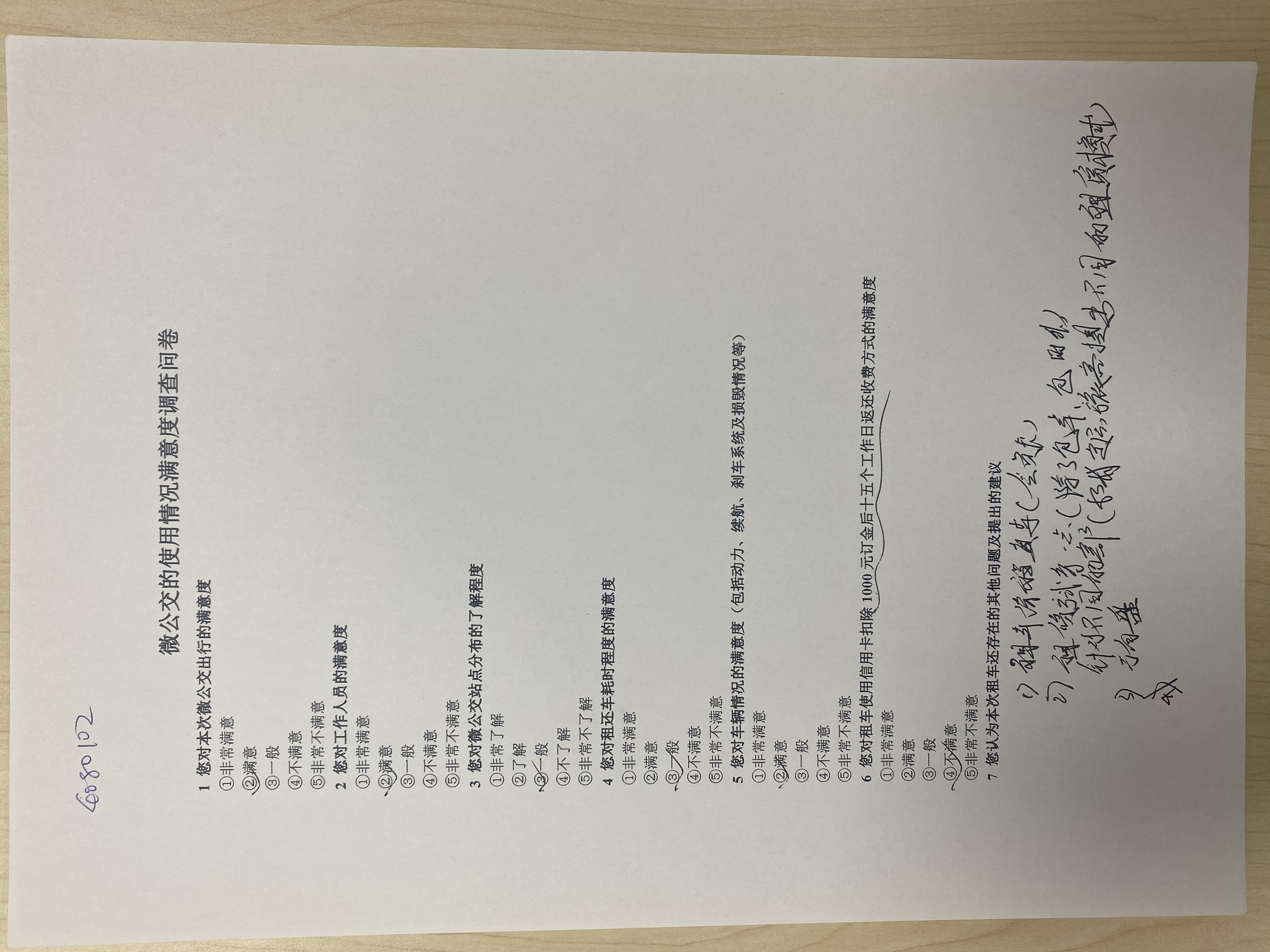

Supplement: S1 File — (ZIP) [file pone.0263476.s001.zip › S1 Questionnaires/Satisfaction survey-Sample 2.jpeg]

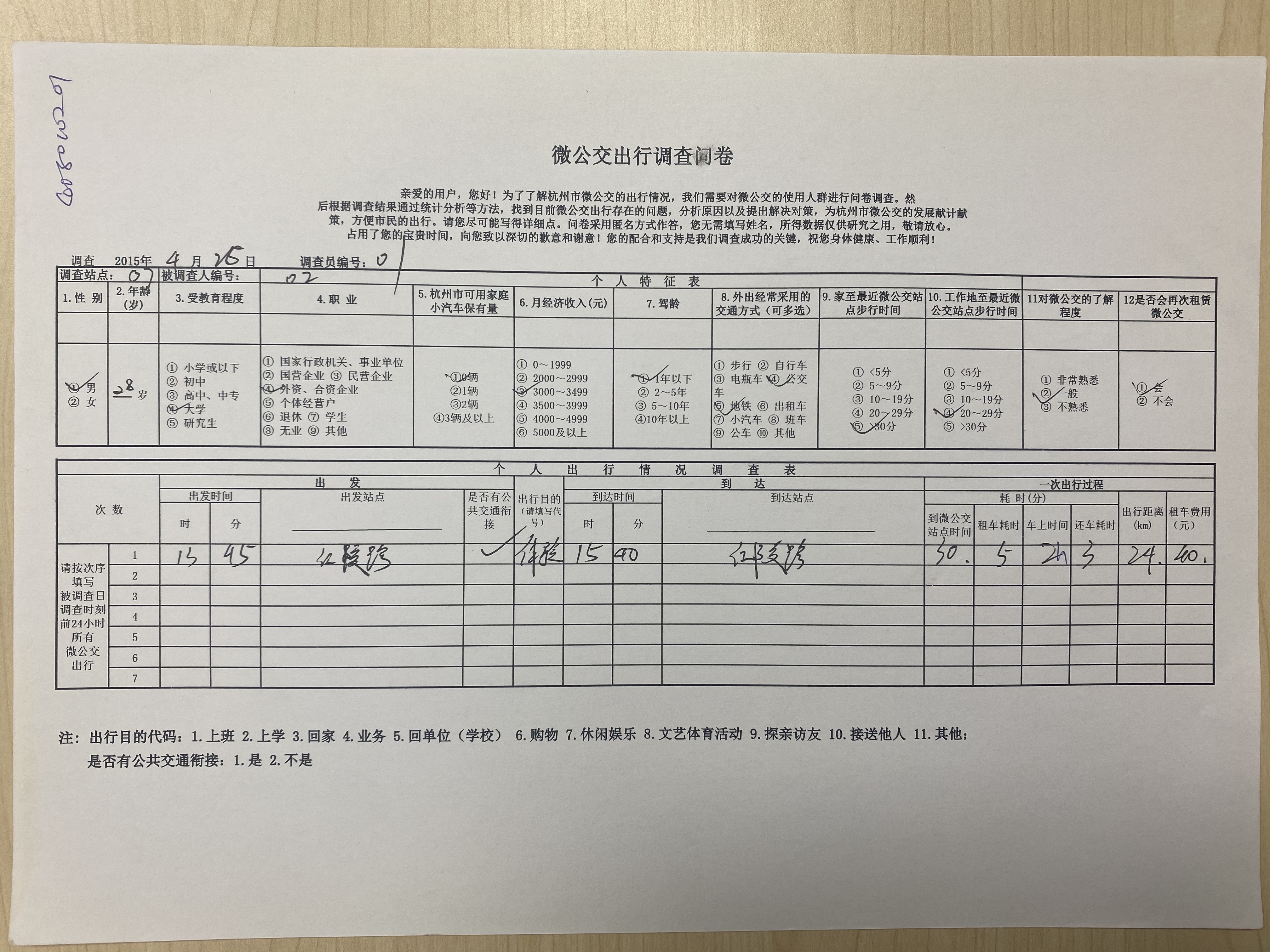

Supplement: S1 File — (ZIP) [file pone.0263476.s001.zip › S1 Questionnaires/PT survey-Sample 2.jpeg]

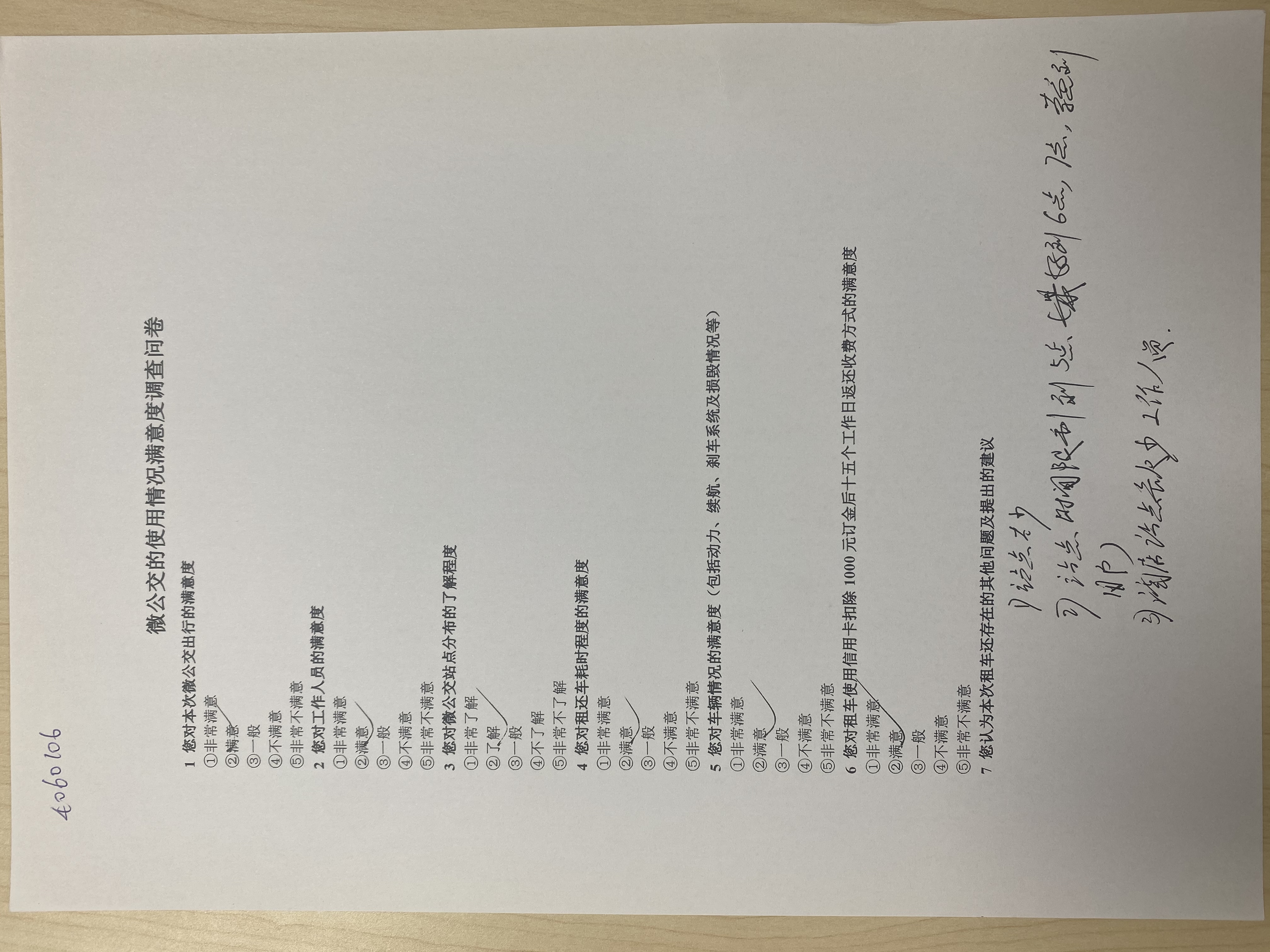

Supplement: S1 File — (ZIP) [file pone.0263476.s001.zip › S1 Questionnaires/Satisfaction survey-Sample 1.jpeg]
